# The *TaWRKY22*–*TaCOPT3D* Pathway Governs Cadmium Uptake in Wheat

**DOI:** 10.3390/ijms231810379

**Published:** 2022-09-08

**Authors:** Xiaojuan Liu, Hongcheng Wang, Fang He, Xuye Du, Mingjian Ren, Yinguang Bao

**Affiliations:** 1 College of Agriculture, Guizhou University, Guiyang 550004, China; 2 School of Life Sciences, Guizhou Normal University, Guiyang 550025, China; 3 State Key Laboratory of Crop Biology, Agronomy College, Shandong Agricultural University, Tai’an 271000, China

**Keywords:** wheat, *TaCOPT3D*, Cd uptake, *TaWRKY22*, transcriptional regulation

## Abstract

Cadmium (Cd) is a heavy metal nonessential for plants; this toxic metal accumulation in crops has significant adverse effects on human health. The crosstalk between copper (Cu) and Cd has been reported; however, the molecular mechanisms remain unknown. The present study investigated the function of wheat *Cu transporter 3D* (*TaCOPT3D*) in Cd tolerance. The *TaCOPT3D* transcripts significantly accumulated in wheat roots under Cd stress. Furthermore, *TaCOPT3D*-overexpressing lines were compared to wildtype (WT) plants to test the role of *TaCOPT3D* in Cd stress response. Under 20 mM Cd treatment, *TaCOPT3D*-overexpressing lines exhibited more biomass and lower root, shoot, and grain Cd accumulation than the WT plants. In addition, overexpression of *TaCOPT3D* decreased the reactive oxygen species (ROS) levels and increased the active antioxidant enzymes under Cd conditions. Moreover, the transcription factor (TF) *TaWRKY22*, which targeted the *TaCOPT3D* promoter, was identified in the regulatory pathway of *TaCOPT3D* under Cd stress. Taken together, these results show that *TaCOPT3D* plays an important role in regulating plant adaptation to cadmium stress through bound by TaWRKY22. These findings suggest that *TaCOPT3D* is a potential candidate for decreasing Cd accumulation in wheat through genetic engineering.

## 1. Introduction

Cadmium (Cd) is a nonessential element for plants and has become one of the most toxic pollutants in water and soil worldwide [1]. Cd interferes with many metabolic and physiological processes in plants, such as disrupting electron transport, decreasing chlorophyll content, causing cell death, and inhibiting nutrient absorption and distribution [2,3]. Excess Cd inhibits plant growth and development in agricultural production and reduces crop yield and quality [4]. Additionally, Cd accumulates in the edible parts of plants poses a potential risk to humans through the food chain [5]. Cultivars with low Cd accumulation capacity can be used to ensure food safety. Moreover, it is crucial to recognize candidates related to Cd uptake and transport to reduce Cd entry/transfer to the food chain.

Several studies have progressed on the molecular aspects of Cd transporters in plants [6,7]. Cd enters root cells via a complex pathway, gets absorbed by the roots, and finally gets translocated to the shoots. However, there is no specific transporter for Cd uptake and transport [8]. As such, transporters responsible for the uptake of essential elements are involved in Cd transport, including iron-regulated transporters (IRTs), zinc-regulated transporter/IRT-like proteins (ZIPs), natural resistance-associated macrophage proteins (NRAMPs), and heavy-metal ATPases (HMAs) [9,10]. Cd has a similar structure to several necessary elements (such as Fe, Mn, and Zn) and can replace these elements in the enzyme [11]. Importantly, enzyme activity is destroyed when Cd enters the cells, leading to growth inhibition, metabolic abnormality, and death [12]. In addition, Cd entering plants causes the production of reactive oxygen species (ROS) that damage protein and DNA, leading to oxidative stress [13]. Under Cd stress conditions, the activity of antioxidant enzymes, including peroxidase (POD), superoxide dismutase (SOD), hydrogen oxidation enzymes (CAT), and glutathione reductase (GR), is increased, thereby enhancing the scavenging of intracellular ROS and reducing oxidative damage [14].

Copper transporter (COPT) is known to perform Cu acquisition and transport in eukaryotes [15]. The functions of the members of the COPT are well known to include the regulation of Cu transport and homeostasis in *Arabidopsis* [16]. Several COPTs transport ions other than Cu. For example, *Arabidopsis* COPT2 is involved in the uptake of Au, while COPT5 participates in response to Fe deficiency [17,18]. In rice, the expression patterns of COPT are influenced by Fe, Mn, or Zn [19]. These works suggest the role of COPT in the uptake and transport of multiple ions. However, the functions of *COPT* in Cd stress response are unknown. In a previous study, we found that the gene *AetCOPT3* in *Aegilops tauschii* was significantly upregulated under cadmium stress [20]. However, the function of *COPT3* in wheat remains unclear.

The current study identified the wheat COPT gene *Cu transporter 3D* (*TaCOPT3D*) under Cd stress. Transcription factor TaWRKY22 binds to the promoter of *TaCOPT3D* and regulates its expression. *TaCOPT3D* overexpression increased Cd content in root tissues but decreased Cd content in grain. Meanwhile, the transgenic wheat showed a high expression of *TaHMA3* upon Cd exposure. Thus, the study’s findings support the role of *TaCOPT3D* under Cd stress and the associated mechanisms, which may provide the foundation for the breeding of Cd-resistant wheat with low Cd levels in the edible parts.

## 2. Results

### 2.1. TaCOPT3D Expression in Wheat under Cadmium Stress

**Figure 1:**
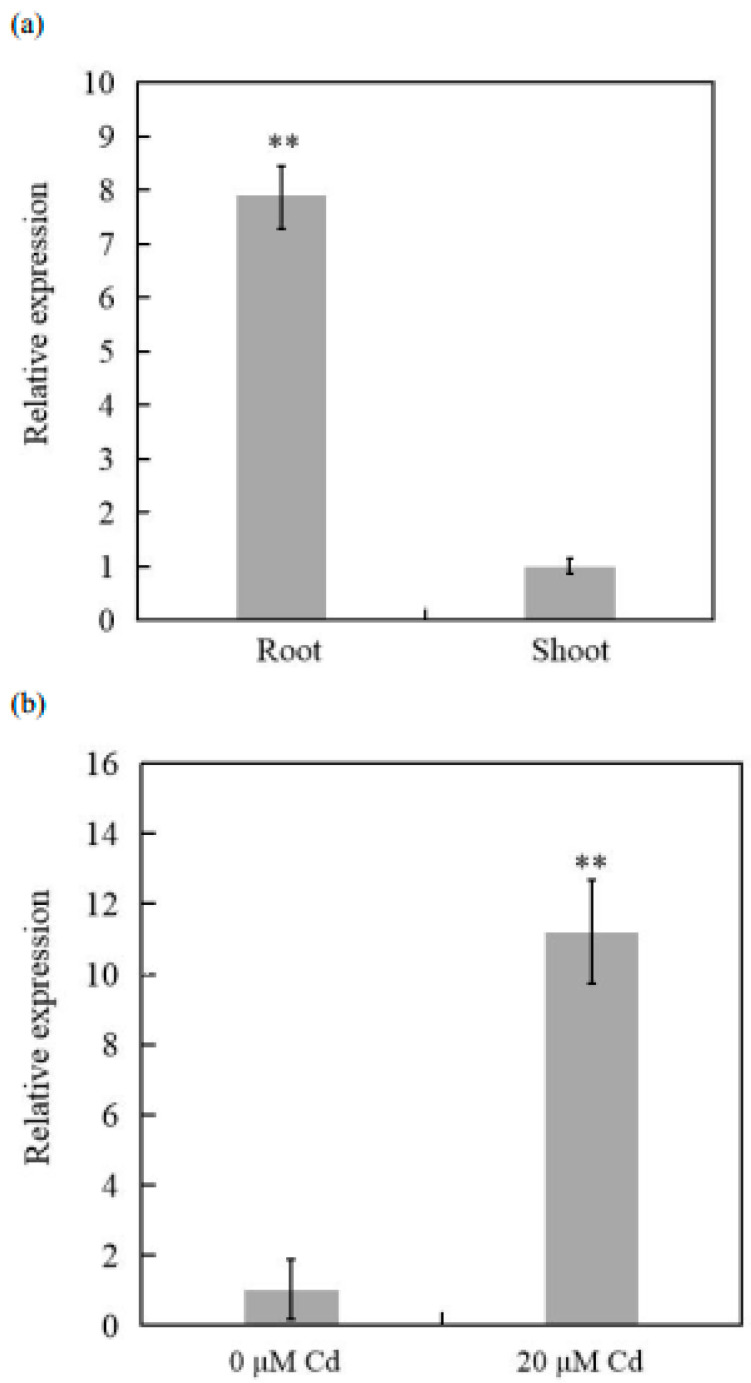
(**a**) *TaCOPT3D* specifically expressed in the root tissue of wheat. (**b**) The expression of *TaCOPT3D* in the root tissue was highly induced by Cd stress. ** Significant differences at *p* ≤ 0.01.

Sequence alignment results showed that the DNA sequences of these three *COPT3* genes were highly similar, but three bases of *TaCOPT3D* located at the 5’ end were missing (Figure S1). Then, *TaCOPT3D*-specific primers for RT-qPCR analysis were designed on the basis of sequence variation (Supplementary Table S1). RT-qPCR revealed that *TaCOPT3D* was particularly expressed in the root tissue of wheat (Figure 1a). In addition, Cd exposure significantly increased the *TaCOPT3D* transcript levels in wheat (Figure 1b).

### 2.2. Subcellular Localization of TaCOPT3D

**Figure 2:**
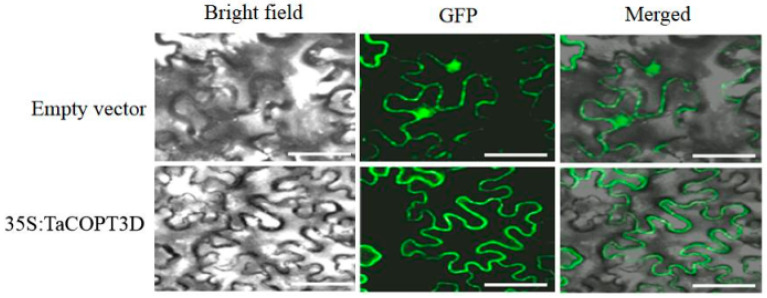
Subcellular localization of TaCOPT3D in tobacco epidermal cells. Scale bars: 10 μM.

The TaCOPT3D was fused into the 3′ region of the GFP gene under the control of a CaMV 35S promoter in the vector to produce the TaCOPT3D–GFP fusion construct. The fusion vector was transiently expressed in the tobacco leaves by *Agrobacterium*-mediated transformation to analyze the protein expression. TaCOPT3D–GFP was localized mainly in the membrane (Figure 2), suggesting that TaCOPT3D probably functioned in the cell membrane. 

### 2.3. Overexpression of TaCOPT3D in Wheat Increased Cd Tolerance 

We created transgenic wheat plants overexpressing *TaCOPT3D* to evaluate the function of *TaCOPT3D* in regulating Cd tolerance. A maize *Ubiquitin* (*Ubi*) promoter-driven binary vector was generated (Supplementary Figure S1a). Two independent transgenic lines (OE1 and OE2) were derived (Supplementary Figure S1b). The expression of TaCOPT3D in transgenic lines was further investigated, and the *TaCOPT3D*-overexpression lines clearly increased *TaCOPT3D* expression to about twofold compared with WT (Supplementary Figure S1c).

**Figure 3:**
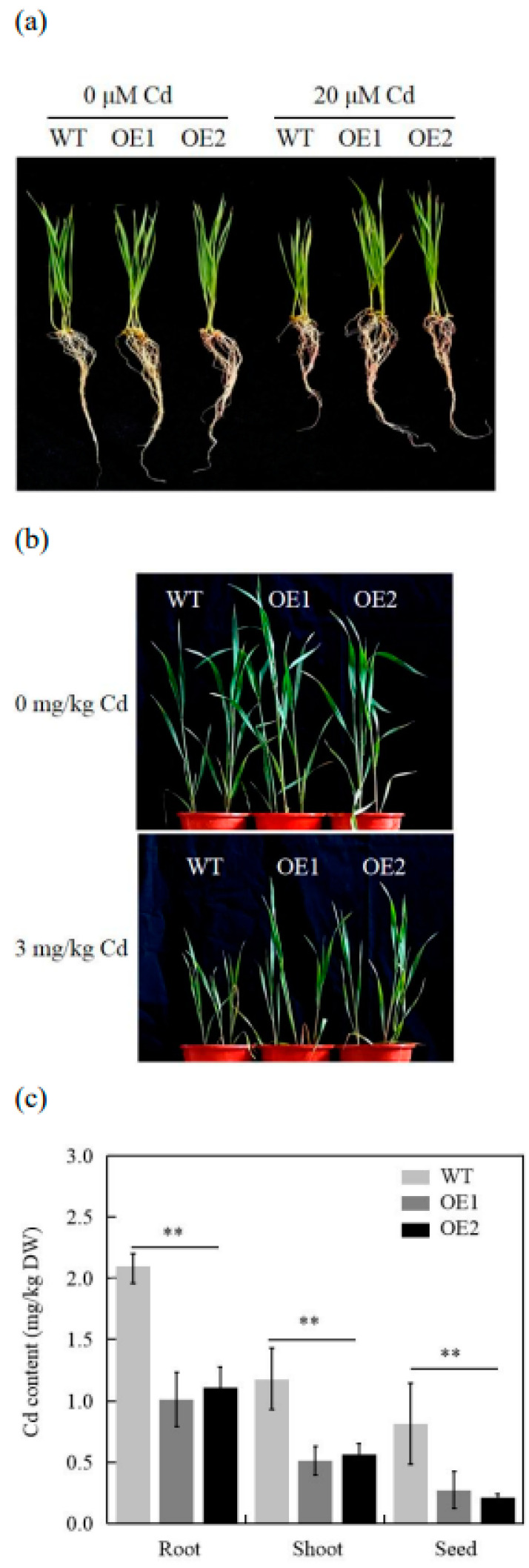
(**a**) Phenotype of WT and transgenic plants under hydroponic conditions. (**b**) Phenotype of WT and transgenic plants under pot conditions. (**c**) Cd concentration in roots, shoots, and grains of the WT and transgenic plants. ** Significant differences at *p* ≤ 0.01.

Furthermore, Cd tolerance in wheat plants was studied through hydroponic and pot experiments to assess Cd tolerance due to the changes in *TaCOPT3D* expression. Under normal conditions, no evident phenotypic dissimilarities were observed between the WT and the transgenic lines (Figure 3a,b). In the hydroponic test using 20 mM Cd, the transgenic lines and the WT demonstrated growth retardation. However, the transgenic lines exhibited improved growth compared to the WT (Figure 3a). The plants were exposed to Cd stress during the vegetative and reproductive stages of growth in a pot experiment (Figure 3b). The WT and transgenic lines appeared similar under normal conditions. Nevertheless, the *TaCOPT3D*-overexpressing lines displayed higher Cd tolerance than the WT under Cd stress (Figure 3b). The comparison of the root, shoot, and grain Cd of WT and transgenic lines after Cd treatment demonstrated that the Cd content was lower for the transgenic lines than the WT line in these tissues (Figure 3c).

These results collectively propose *TaCPOT3D* as a positive regulator of Cd tolerance in wheat.

### 2.4. Overexpression of TaCOPT3D Enhanced the Antioxidant Capacity in Transgenic Wheat under Cd Stress

**Figure 4:**
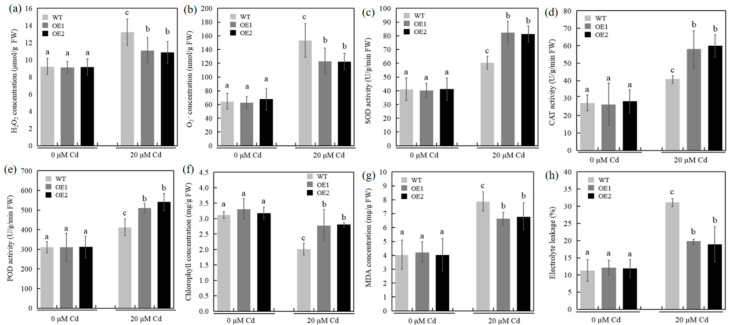
(**a**) H_2_O_2_, (**b**) O_2_^•−^, (**c**) SOD, (**d**) CAT, (**e**) POD, (**f**) chlorophyll, (**g**) MDA, and (**h**) electrolyte leakage in WT and transgenic plants. H_2_O_2_, O_2_^•−^, SOD, CAT, POD, MDA, and electrolyte leakage were measured in the root tissue; chlorophyll concentrations were measured in the leaves. The same letters indicate no significant differences, while different letters indicate a statistically significant difference of the values (*p* ≤ 0.05).

Abiotic stress induces ROS accumulation in plants. The levels of H_2_O_2_ and O_2_^•−^ accumulation in the WT and transgenic line root tissues under hydroponic conditions were investigated. The analysis showed no significant differences in the root H_2_O_2_ and O_2_^•−^ levels between the WT and transgenic lines without Cd stress (Figure 4a,b). Meanwhile, Cd stress increased H_2_O_2_ and O_2_^•−^ levels in both plants; however, the transgenic line H_2_O_2_ and O_2_^•−^ levels were significantly lower than the WT (Figure 4a,b).

We further examined the activities of antioxidant enzymes (SOD, CAT, and POD) between the transgenic and WT lines. Figure 4c–e display similar antioxidant enzyme activities in the WT and transgenic lines under normal conditions. Meanwhile, Cd stress increased antioxidant enzyme activities in the WT and transgenic lines, with significantly higher activities in the transgenic lines than in WT.

Physiological indicators, including malondialdehyde (MDA) concentration, electrolyte leakage, and chlorophyll concentration, were examined. The overexpression of *TaCOPT3D* increased chlorophyll concentration (Figure 4f). Analysis of the correlation between chlorophyll content and net photosynthesis suggested that the transgenic plants retained photosynthesis under Cd stress. MDA content and electrolyte leakage were closely correlated with the degree of cell membrane damage under abiotic stress [21]. The MDA content increased in all samples of the study under Cd stress compared with those under normal situations; however, upon Cd exposure, the transgenic lines had significantly lower MDA content than WT plants (Figure 4g). In addition, the electrolyte leakage was lower in transgenic wheat seedlings than the WT seedlings under Cd stress (Figure 4h). Thus, *TaCOPT3D* overexpression increased Cd tolerance and inhibited Cd accumulation in wheat. Additionally, the lower MDA content and electrolyte leakage of the transgenic plants upon Cd exposure reflected a lower degree of damage to the plant cell membranes.

### 2.5. TaCPOT3D Influences the Cd^2+^ Flux in Wheat Roots

**Figure 5:**
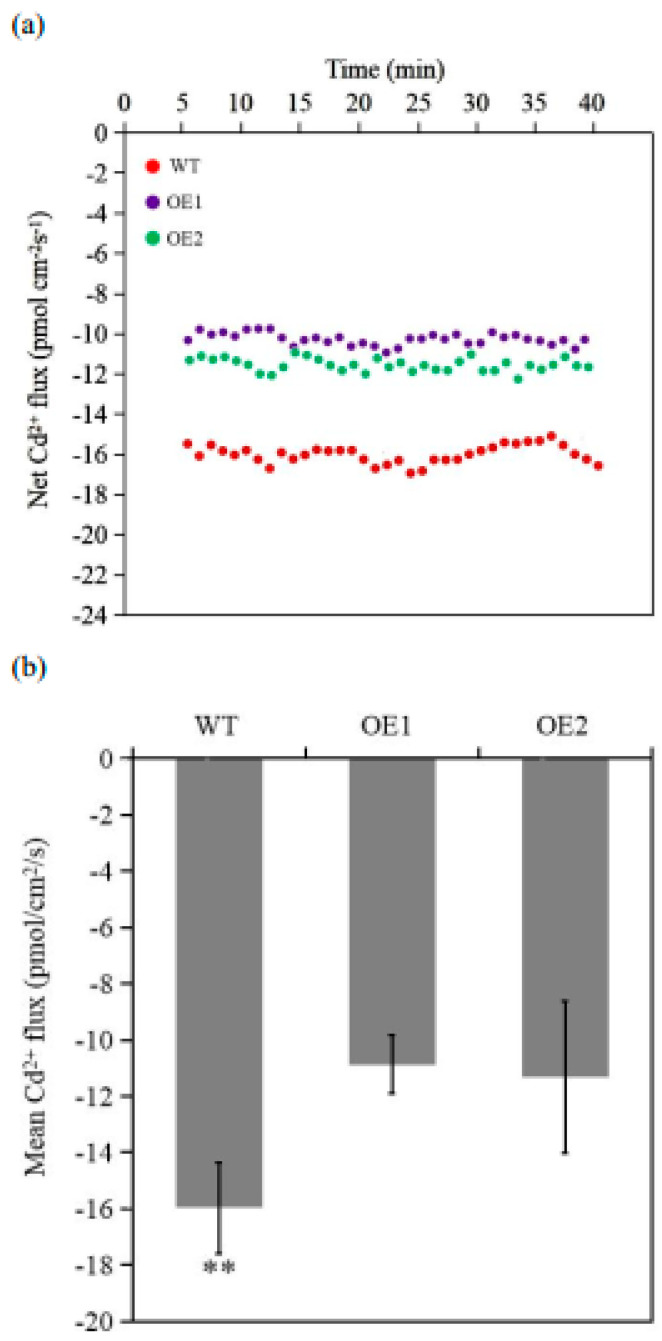
(**a**) Transient Cd^2+^ flux of WT and transgenic wheat root tissues under Cd condition. (**b**) Average Cd^2+^ flux of WT and transgenic wheat root tissues under Cd condition. ** Significant differences at *p* ≤ 0.01.

Furthermore, a noninvasive micro-test technique was used to record the transient Cd^2+^ flux. The Cd^2+^ influx was less in *TaCPOT3D*-overexpressing lines than WT under Cd stress (Figure 5a,b), indicating decreased Cd enrichment with *TaCPOT3D* overexpression via net Cd^2+^ flux inhibition. 

### 2.6. TaWRKY22 Binds to TaCPOT3D Promoter

**Figure 6:**
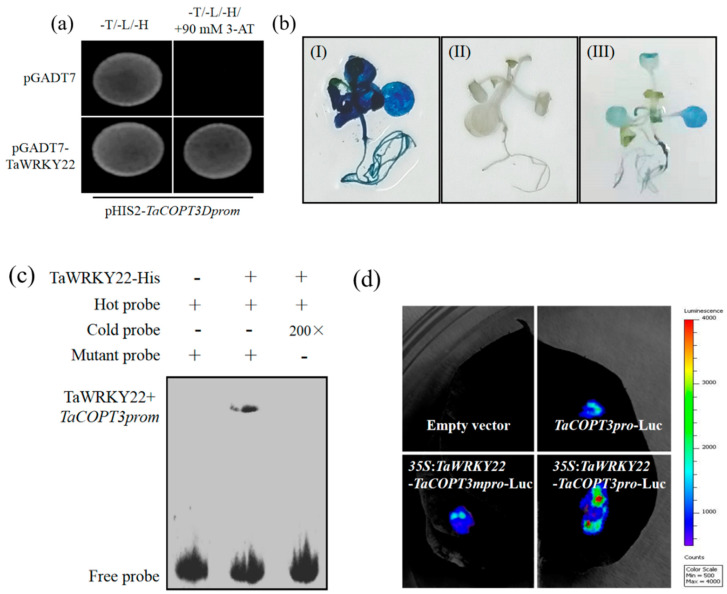
(**a**) Yeast one-hybrid assays; (**b**) Identification of TaWRKY22 binds to W-box of *TaCOPT3D* promoter. (I): *Arabidopsis* expression of *TaCOPT3D*-GUS; (II): *Arabidopsis* expression of *TaWRKY22*; (III): *Arabidopsis* co-expression of *TaCOPT3D*-GUS and *TaWRKY22*; (**c**) EMSA assay showing that TaWRKY22 fusion protein directly bound to the *TaCOPT3D* promoter in vitro; (**d**) Transient expression assays showing that *TaWRKY22* regulates the expression of *TaCOPT3D*.

Furthermore, a cDNA library was generated using the Cd-treated wheat samples, and a yeast one-hybrid (Y1H) assay was performed using *TaCOPT3D* promoter as bait to identify *TaCOPT3D* regulation. Twenty-seven positive colonies were sequenced, and a WRKY TF was characterized. A BLAST search for this sequence (www.ncbi.nlm.nih.gov/BLAST, accessed on 16 February 2021) revealed *TaWRKY22* as the TF. The Y1H experiment was performed to confirm the effects (Figure 6a).

Next, we confirmed that *TaWRKY22* acted as a transcription factor using transgenic *Arabidopsis*. Here, pROKII-*TaWRKY22* acted as an effector, and pCAMBIA1301-*TaCOPT3Dprom* worked as a reporter. The GUS intensity in *TaCOPT3Dprom* transgenic *Arabidopsis* was significantly stronger than the co-transformed *Arabidopsis* with *TaWRKY22* and *TaCOPT3Dprom* (Figure 6b). EMSA was further conducted to validate the TaWRKY22-binding ability of *TaCOPT3Dprom*. As indicated in Figure 6c, the TaWRKY22–*TaCOPT3Dprom* complex was observed. Comparison of the in vivo and in vitro results suggested that TaWRKY22 is bound to the *TaCOPT3Dprom*.

A transcriptional activity assay in tobacco leaves was performed to analyze whether TaWRKT22 activated *TaCOPT3D* transcription. As indicated in Figure 6d, TaWRKY22 promoted the expression of the Luc gene under the control of the *TaCOPT3D* promoter. These observations indicate that TaWRKT22 directly activates the *TaCOPT3D* promoter through in vivo transcription.

## 3. Discussion

Studies have extensively studied the *COPT* genes associated with Cu, Fe, and Zn absorption. However, it is unclear how *COPT* genes act in response to Cd stress in plants. The current research explored *TaCOPT3D* in wheat under Cd stress. We further found that *TaCOPT3D* was strongly regulated by the *TaWRKY22* TF. Thus, the results of this study preliminarily clarified the wheat Cd stress transcriptional pathway *TaWRKY22*–*TaCOPT3D*.

The uptake, transportation, and accumulation of metal ions in plants depend on ion transporters [22]. The NRAMP, ATP-binding cassette (ABC) transporter, HMAs, yellow-stripe-like (YSL) proteins, and ZIP metal transporters have been identified as putative metal ion transporters [23]. Transporters capable of binding metal ions and the transport of ions through ion channels are located on the cellular membrane of each plant cell [24]; plants have distinct Cd transporters. Previous reports have demonstrated that Cd entry into plant cells occurs via transporters for other divalent metal ions [8]. A previous study reported that NRAMP5 in rice (*OsNRAMP5*) functions as a metal transporter for Mn and Cd uptake [21,25]. The overexpression of *OsNRAMP5* blocked the radial transport of Cd from the epidermis to the xylem and reduced cadmium accumulation in rice grains [25]. *TpNRAMP5* from *Triticum polonicum* enhanced the accumulation of Cd, Co, and Mn, but not Zn and Fe [8]. COPT acts as a high-affinity Cu transporter in plants, and some COPTs are also involved in the Cd response [19]. *Arabidopsis thaliana COPT5* mutants (*copt5*) are more sensitive to Cd stress than WT plants, and ethylene biosynthesis diminishes in the presence of Cd [26]. Yuan et al. reported seven members of *COPTs* in rice, all of which were characterized for their functions in Cu transport [19]. In addition, these COPTs cannot mediate Fe and Zn uptake, suggesting that COPTs facilitate the selective absorption of Cu, Fe, and Zn [19]. *TaCOPT3D* overexpression in this study increased the Cd tolerance of wheat. In addition, Cd absorption diminished further under exogenous Cu application. In yeast cells, Cd was shown to modify Cu deficiency responses [27]. In rice, Cu could effectively alleviate the stress induced by Cd and increase the rice biomass and ripening rate; however, 10 μM CuSO_4_ significantly increased the Cd concentration in rice grains [28]. Similar results in *A. thaliana* showed that the Cd content in shoot tissue significantly increased when grown in a medium supplemented with 0.1 mM Cu [26]. 

Transcriptional regulation may be an adaptation to respond to different levels of abiotic and biotic stresses [29]. Plants often respond to environmental stimuli by activating specific TFs, and the TFs then bind to the promoter sequences of target genes to initiate the transcription of downstream genes to respond to environmental changes [30]. In the present work, Y1H screening using the *TaCOPT3D* promoter as bait was conducted to identify a TF that targeted the *TaCOPT3D* promoter (Figure 6a). The results demonstrated that TaWRKY22 could activate the gene expression of the *TaCOPT3D* promoter (Figure 6c,d). WRKY proteins constitute a large family of TFs in plants involved in plant growth, development, and responses to biotic and abiotic stresses [31]. The WRKY TF binds explicitly to the W-box or W-box-like elements containing the TGAC core sequence [32]. Numerous transcriptional regulation pathways have been shown to serve as important mechanisms in response to heavy-metal stress in plants [32,33,34]. WRKY13 activates PDR8 expression to positively regulate Cd tolerance in *Arabidopsis* [31]. *Arabidopsis* A4 heat-shock TF *HsfA4a* regulates Cd tolerance by activating the expression of the metallothionein gene [35]. In addition, *OsWRKY22* promotes aluminum (Al) tolerance via the activation of *OsFRDL4* expression in rice [30]. The present work using overexpressing *Arabidopsis* demonstrated that TaWRKY22 bound to the W-box in the *TaCOPT3D* promoter (Figure 6b). *TaWRKY22*–*TaCOPT3D* constitutes a regulatory pathway involved in Cd response in wheat.

## 4. Materials and Methods

### 4.1. Plant Materials and Treatments

The wheat cultivar Bobwhite, as the wildtype (WT) line, was used for genetic engineering. For the hydroponic experiment, the seeds of Bobwhite were germinated in ddH_2_O, transferred to Hoagland’s nutrient solution, and maintained in a growth chamber with 25/20 °C day/night temperatures and a photoperiod of 16 h/day using photosynthetically active radiation. At the trefoil stage, the wheat seedlings were treated with 20 mM Cd (CdCl_2_·5 H_2_O), while seedlings maintained with no treatment were used as the control (0 mM Cd). The seedlings were collected at different timepoints (0 h, 1 h, 3 h, 6 h, 12 h, 24 h, and 14 days) after the treatment, immediately frozen in liquid nitrogen, and stored at −80 °C for further analysis of enzyme activity, gene expression, and ion content. For the pot experiment, germinated seeds were maintained at 4 °C for 14 days, transplanted into pots, and maintained in a phytotron with a 12 h photoperiod using a cool white fluorescent light and an indoor temperature of 26 °C. These pots were further divided into two groups; one had 1 kg of peat soil alone (control), and the second had 1 kg peat soil with 1 mg Cd (CdCl_2_·5 H_2_O; experimental group). Before planting wheat, 200 g of compound fertilizer (15% nitrogen, 10% phosphorus, and 15% potassium) was added to each pot. The plants were irrigated with 20 L of water every 2 weeks until wholly ripened. Finally, 10 seedlings were selected per treatment per line to determine seed Cd content.

### 4.2. Cloning of Coding Sequence of TaCOPT3D

The total RNA from the root and shoot tissue was extracted using an RNApure Plant Kit (CwBio, Beijing, China), following the manufacturer’s instructions. A total of 2 μg of RNA was employed to synthesize the first-strand cDNA using a SuperRT cDNA Synthesis Kit (CwBio, Beijing, China). *TaCOPT3D* was cloned from cDNA using primers designed on the basis of the reference sequence of TraseCS3D02G306300. The high–fidelity DNA polymerase Ex Taq (Takara, Dalian, China) was used to amplify all the required gene products. The amplified fragment was cloned into pMD18-T vector (Takara, Dalian, China) and sequenced. At least three independent clones were sequenced.

### 4.3. Analysis of Gene Expression

Multiple alignment of the coding sequences of *TaCOPT3A* (TraesCS3A02G296400), *TaCOPT3B* (TraesCS3B02G340700), and *TaCOPT3D* (TraesCS3D02G306300) was performed using DNAMAN software. Primers for real-time quantitative PCR (RT-qPCR) were designed on the basis of specific regions identified from the multiple sequence alignments of *TaCOPT3A*, *TaCOPT3B*, and *TaCOPT3D*. The expression of the genes was analyzed using an UltraSYBR One–Step RT–qPCR Kit (CwBio, Beijing, China) in a real–time polymerase chain reaction (PCR) system (LightCycler 96, Roche, Basel, Switzerland). The wheat *β-actin* was used as an internal control, and the relative expression levels of genes were calculated using the 2^−^^∆∆^^CT^ method [36].

### 4.4. Gene Cloning and Plant Transformation

The open reading frame (ORF) of *TaCOPT3D* was amplified and cloned into the pCambia3300 vector at the *BamH* I and *Kpn* I restriction sites to obtain the *Ubi*:*TaCOPT3D* construct. All binary vectors harboring the desired constructs were transferred into strain EHA105 and transformed into the wheat cultivar Bobwhite using *Agrobacterium*-mediated transformation [37].

Promoter of *TaCOPT3D* were cloned into pCAMBIA1301 to drive β-glucuronidase (GUS), generating the pCAMBIA1301-*TaCOPT3Dprom*-box. The *TaWRKY22* was cloned into the pROKII vector, generating pROKII-TaWRKY22. Then pROKII-*TaWRKY22* and pCAMBIA1301-*TaCOPT3Dprom*-box were transformed into *Arabidopsis*, respectively. To investigate transient co-expression of the *TaWRKY22* and *TaCOPT3Dprom* pCAMBIA1301-*TaCOPT3Dprom*-box and pROKII-*TaWRKY22* were co-transiently transformed into *Arabidopsis*. The *Arabidopsis* transformation was carried out using the *Agrobacterium tumefaciens*-mediated method [26].

### 4.5. Detection of Antioxidant Enzyme Activity

The determination of antioxidant enzymes, including superoxide dismutase (SOD), peroxidase (POD), and catalase (CAT), was conducted using test kits (Solarbio, Beijing, China) according to the manufacturer’s instructions.

### 4.6. Measurements of Cd^2+^ Flux

The WT and transgenic wheat plants were used for ion flux measurements. Trefoil-stage wheat seedlings were treated with Hoagland’s nutrient solution or 20 mM Cd within Hoagland’s nutrient solution for 24 h. The net Cd^2+^ flux was measured in wheat root meristematic tissues using noninvasive micro-test technology as described by Zhang et al. [38].

### 4.7. Measurement of Cd Contents

The Cd contents of seedlings and grains were measured using inductively coupled plasma mass spectroscopy, according to Zhang et al. [38]. The Cd fluorescence in root tissues was visualized using the Leadmium Green fluorescent probe (Invitrogen, Carlsbad, CA, USA) according to the manufacturer’s protocol.

### 4.8. Yeast One-Hybrid (Y1H) Assay

The cDNA library was constructed in our previous work [37]. Y1H library screening was performed according to Lin et al. [39]. The Y1H assay was conducted in the yeast Y187 strain using the MATCHMAKER One Hybrid System (Clontech, CA, USA), following the manufacturer’s instructions. The *TaCPOT3D* promoter was inserted into the pHIS2 reporter vector to obtain pHIS2-*TaCPOT3Dpro*. The pHIS2-*TaCPOT3Dpro* and pGADT7-TaWRKY22 plasmids were co-transformed into Y187, and the culture was spread on a synthetically defined double drop-out medium lacking tryptophan (Trp) and leucine (Leu). The transformant was tested on a medium lacking leucine (Leu), tryptophan (Trp), and histidine (His) supplemented with 90 mM 3-amino-1,2,4-triazole (3-AT) for 3 days.

### 4.9. Electrophoretic Mobility Shift Assay (EMSA)

An oligonucleotide sequence of the *TaCOPT3D* promoter was produced and biotin-labeled at the 3’ end by Sangon (Shanghai, China). The *TaWRKY22* coding sequence was cloned into the pGEX4T-1 vector and subsequently subjected to the EMSA assay using the LightShift Chemiluminescent EMSA Kit (Pierce, Rockford, IL, USA).

### 4.10. Transient Expression Assays in Tobacco Leaves

Transient expression assays were performed in tobacco leaves as described by Shang et al. [40]. *TaCPOT3Dprom* was cloned into pGreenII 0800-LUC to obtain the reporter vector, and *TaWRKY22* ORF was cloned into pGreenII 62-SK to create the effector vector. Expression of luciferase (LUC) in the transformed cells was detected by capturing the images using a NightOWL II LB983 apparatus (Berthold, Germany).

### 4.11. Statistical Analysis

Student’s *t*-test was used to compare the mean values and detect statistically significant differences. Three independent biological replicates were maintained per experiment. A *p*-value less than 0.05 was considered statistically significant.

### 4.12. Primers

Supplementary Table S1 shows all primers used in this study.

## 5. Conclusions

The current study revealed the function of *TaCOPT3D* in the Cd response of wheat. The overexpression of *TaCOPT3D* activated the ROS-scavenging system, which resulted in enhanced Cd tolerance. Moreover, a WRKY TF, *TaWRKY22*, was found to regulate the expression of *TaCOPT3D* by binding the W-box in the *TaCOPT3D* promoter. Thus, *TaCOPT3D* may be a candidate gene for producing safe wheat grains in Cd-contaminated soil.

## Data Availability

The data presented in this study are available on request from the corresponding author.

## Supplementary Materials

The following supporting information can be downloaded at https://www.mdpi.com/article/10.3390/ijms231810379/s1.
