# Supplementary material for: The TaWRKY22–TaCOPT3D Pathway Governs Cadmium Uptake in Wheat
Source: Int J Mol Sci. 2022 Sep 8;23(18):10379. doi: 10.3390/ijms231810379 (PMC9499326; doi:10.3390/ijms231810379)

Supplementary Figure Legends

Figure S1. (a) Schematics of the plant expression vector used in the wheat transformation. (b) Identification of transgenic wheat lines using PCR. (c) Gene expression of *TaCOPT3D* in WT and transgenic lines. **indicates a significant difference at $P \leq 0.01$.

Supplementary Figure S1

(a)

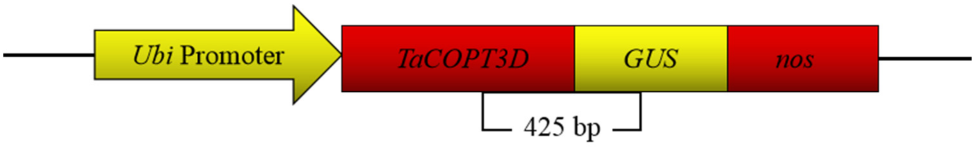

(b)

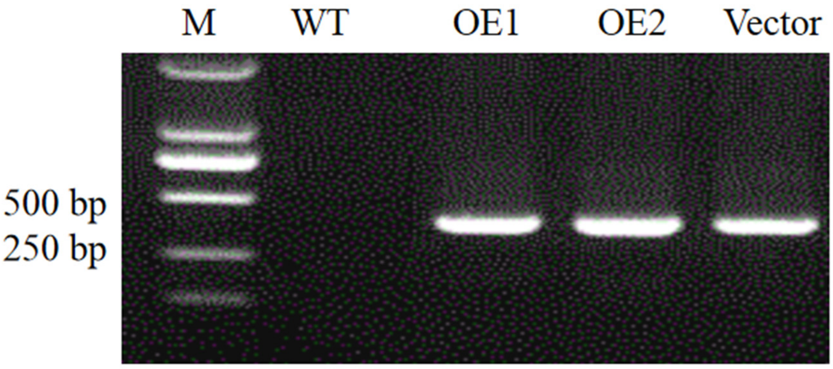

(c)

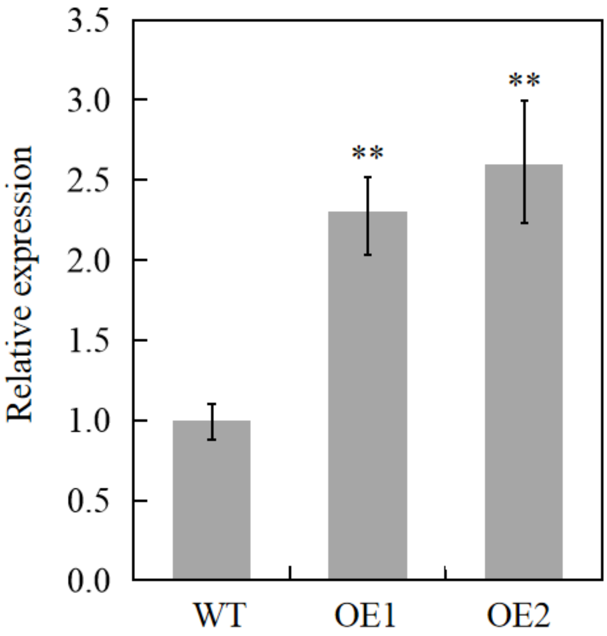

Supplement: Supplementary file 1 [file ijms-23-10379-s001.zip › ijms-1862649-supplementary/ijms-1862649-supplementary/Supplementary Figure.pdf]
