# Supplementary material for: The TaWRKY22–TaCOPT3D Pathway Governs Cadmium Uptake in Wheat
Source: Int J Mol Sci. 2022 Sep 8;23(18):10379. doi: 10.3390/ijms231810379 (PMC9499326; doi:10.3390/ijms231810379)
Supplement: Supplementary file 1 [file ijms-23-10379-s001.zip › ijms-1862649-supplementary/ijms-1862649-supplementary/Supplementary Table.pdf]

Table S1. Primers used in the present work.

Primers	Sequences (5'-3')	Description
F1	CTAACGTCATTCA AACTACC	qPCR for *TaCOPT3D*
R1	TTACGGAATGGTC CAACGTACC	
F2	ATCCAGCCCAGGA CCCAACAC	Cloning of *TaCOPT3D*
R2	TAGGCAACGGTAA CTTGCAA	
F3	**GGATCC**ATGGTA AGCTTGACGAC	Generation of vector for wheat transformation and prokaryotic expression. Restriction site of *BamH*I in F3 and *Kpn*I in R3 marked in bold.
R3	**GGTACC**AATAAA CGTTAGCGGCA	
F4	**GGATCC**ATGGTA AGCTTGACGAC	Generation of vector for Y1H assay. Restriction site of *BamH*I in F4 and *Nde*I in R4 marked in bold.
R4	**CATATG**AATAAA CGTTAGCGGCA	
F5	TGCAACTCGGTAG TACCAA	Detecting transgenic wheat expressing *TaCOPT3D*
R5	CTAGGCATGGCTG AATG	
